# *Educational Resource Review:* MSD’s The Steward—Episode 4—Bacterial infections in children

**DOI:** 10.1093/jacamr/dlab065

**Published:** 2021-06-15

**Figure:**
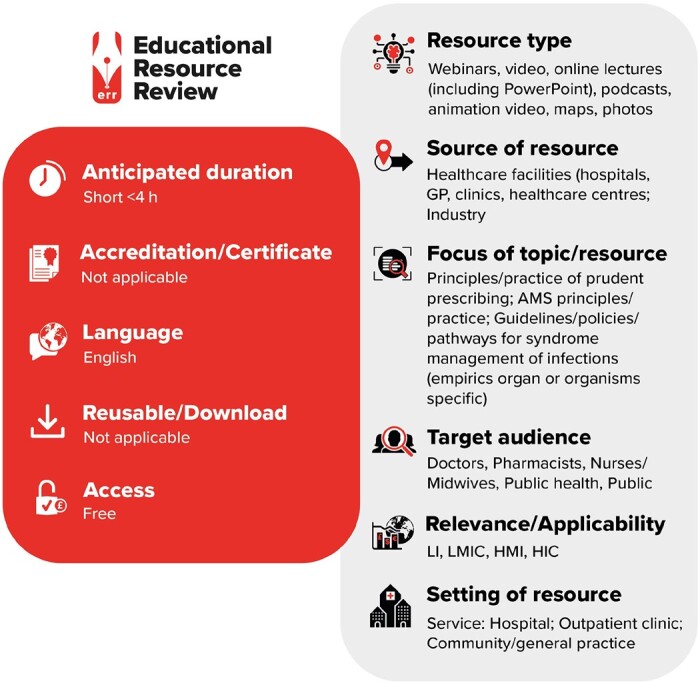


LI, low-income countries; LMIC, low- and middle-income countries; HMI, high- and middle-income countries; HIC, high-income countries.

**Resource web link:** https://www.youtube.com/watch?v=5o0qEXg2DGw (Full classification scheme available at: http://bsac.org.uk/wp-content/uploads/2019/03/Educational-resource-review-classification-scheme.pdf)

**WHO region and country (World Bank):** European Region, England (HIC)

## Peer review commentary

This resource provides an excellent overview of stewardship in paediatrics delivered via a podcast of a discussion between the interviewer from MSD and Dr Sanjay Patel, an expert in paediatric infectious diseases. It is applicable for all healthcare staff involved in paediatric care but also of general interest to interested members of the public. The session is engaging as it covers topical issues, and both the interviewer and interviewee are skilled at delivering this type of session. Reflections on COVID-19, recent publications, the UK paediatric network and current research are discussed.

Its key strength is the involvement of a well-known opinion leader in the field. The weakness is that it is personal opinion and some of the content focuses on practice in England, which may not be transferable to other countries, particularly LMICs. The link to YouTube works correctly.

The presenter is a well-respected clinician from a centre leading the way in UK paediatric stewardship. No suggestions for improvement, but there is potential for further sessions to go into more detail on specific infections and antimicrobial management.

The resource complements other podcasts in the series on antimicrobial stewardship.

The presenter mentions a useful network that could be accessed to learn more about the topic, including the latest work on UK guidelines and pathways: https://uk-pas.co.uk/.

Overall, this is an interesting and engaging session that provides a good introduction to the topic and reflections on current practice but will mostly be useful for UK/European clinical teams.

